# Supplementary material for: A deep hierarchy of predictions enables online meaning extraction in a computational model of human speech comprehension
Source: PLoS Biol. 2023 Mar 22;21(3):e3002046. doi: 10.1371/journal.pbio.3002046 (PMC10079236; doi:10.1371/journal.pbio.3002046)
Supplement: S1 Appendix — (DOCX) [file pbio.3002046.s006.docx]

# Model parameters for lemma generation

1. **Possible states and prior belief (D matrix) for each factor**
2. Context level

**Context** = {‘poker game’, ‘tennis game’, ‘night party’, ‘racing game’}, Nc = 4

D{1} = [1.5 1 1 1]

**Type** = {‘event’, ‘property’}, NTy = 2

D{2} = [1 1]

1. Semantic

**Agent** = {‘card A’, ‘serve’, ‘run’, ‘card J’, ‘neckband’, ‘score’, ‘buzz’}, Na = 7

**Relation** = {‘win’, ‘ruin’, ‘be’}, Nr = 3

**Patient** = {‘tennis’, ‘poker’, ‘game’, ‘evening’, ‘null’}, Np = 5

**Modifier** = {‘sufficient’, ‘unexpected’, ‘not pretty’, ‘not fair’, ‘high volume’, ‘high freq’, ‘null’}, Nm = 7

Prior belief for semantic and syntax factors are calculated by multiplying the higher-level priors with probability mapping matrices defined in the next section.

1. Syntax

For syntax, we treat each epoch differently as if there is one factor syntax{i} for each epoch.

**Syntax**{1-4} = {‘attribute’, ‘subject’, ‘verb’, ‘object’, ‘adjective’}, Nsyn = 5

1. Lemma

**Lemma** = {‘one more’, …, ‘sharp’}, Nw = 20. The matching between lemma and syntax + semantic is defined in Appendix I. Multiple meanings are arranged assuming meaning 1 is the major meaning, meaning 2 & 3 are less likely.

1. **Probabilistic mapping for the generative model**

In all mapping matrices, the first dimension represents the outcome factor, the 2^nd^ and further dimensions represent states of the higher level. All matrices are normalized so that the first dimension add to 1.

1. Context to semantic

For the mapping from context c and sentence type Ty to a semantic factor s (s={ a’, ‘r’, ‘p’, ‘m’}), the model is defined by a 3-D matrix L{s}(Ns, Nc, NTy). Indices refer to the state of the factor, e.g. L{agent}(1, 1, 2) = p(agent=’card A’|context=’poker’, type=’property’).

**Context = ‘poker game’ = 1:**

L{agent}(‘card A’, 1, 1) = L{agent}(‘card A’, 1, 2) = 0.6, L{agent}(‘card J’, 1, 1) = L{agent}(‘card J’, 1, 2) = 0.4

L{relation}(‘win’, 1, 1) = 1, L{relation}(‘be’, 1, 2) = 1

L{patient}(‘poker’, 1, 1) = 1, L{patient}(‘null’, 1, 2) = 1

L{modifier}(‘null’, 1, 1) = 1, L{modifier}(‘sufficient’, 1, 2) = L{modifier}(‘unexpected’, 1, 2) = 0.5

**Context = ‘tennis game’ = 2:**

L{agent}(‘serve’, 2, 1) = L{agent}(‘serve’, 2, 2) = 0.6, L{agent}(‘run, 2, 1) = L{agent}(‘run’, 2, 2) = 0.4

L{relation}(‘win’, 2, 1) = 1, L{relation}(‘be’, 2, 2) = 1

L{patient}(‘tennis’, 2, 1) = 1, L{patient}(‘null’, 2, 2) = 1

L{modifier}(‘null’, 2, 1) = 1, L{modifier}(‘sufficient’, 2, 2) = L{modifier}(‘unexpected’, 2, 2) = 0.5

**Context = ‘night party’ = 3:**

L{agent}(‘neckband’, 3, 1) = L{agent}(‘neckband’, 3, 2) = 0.6, L{agent}(‘buzz’, 3, 1) = L{agent}(‘buzz’, 3, 2) = 0.4

L{relation}(‘ruin’, 3, 1) = 1, L{relation}(‘be’, 3, 2) = 1

L{patient}(‘evening’, 3, 1) = 1, L{patient}(‘null’, 3, 2) = 1

L{modifier}(‘null’, 3, 1) = 1

L{modifier}(‘not pretty’, 3, 2) = L{modifier}(‘high volume’, 3, 2) = L{modifier}(‘high freq’, 3, 2) = 1/3

**Context = ‘racing game’ = 4:**

L{agent}(‘score’, 4, 1) = L{agent}(‘score’, 4, 2) = 0.6, L{agent}(‘buzz’, 4, 1) = L{agent}(‘buzz’, 4, 2) = 0.4

L{relation}(‘ruin’, 4, 1) = 1, L{relation}(‘be’, 4, 2) = 1

L{patient}(‘game’, 4, 1) = 1, L{patient}(‘null’, 4, 2) = 1

L{modifier}(‘null’, 4, 1) = 1

L{modifier}(‘not fair’, 4, 2) = L{modifier}(‘high volume’, 4, 2) = L{modifier}(‘high freq’, 4, 2) = 1/3

1. Sentence type to syntax

For syntax, we define the model Z for each epoch τ separately. Each Z{τ} is a Nsyn x NTy matrix, i.e. 5x2

Z{1}(:, 1) = Z{1}(:, 2) = [1 0 0 0 0]. The first epoch is always ‘attribute’

Z{2}(:, 1) = Z{2}(:, 2) = [0 1 0 0 0]. The second epoch is always a subject

Z{3}(:, 1) = Z{3}(:, 2) = [0 0 1 0 0]. The third epoch is always a verb

Z{4}(:, 1) = [0 0 0 1 0], Z{4}(:, 2) = [0 0 0 0 1]. The fourth epoch could be either an object (sentence type=’event’) or an adjective (Ty=’property’).

Now we can calculate priors for semantic and syntax (matrix multiplication is only demonstrative, not concerning matrix transposing in practice).

Semantic: D{3-6} = L{1-4}*D{1}*D{2}

Syntax: D{7}(:, τ) = Z{τ}*D{2}

1. Semantic to lemma

Because we have a one-to-one correspondence between syntax and semantic, we define the model A with Nsyn independent matrices, each mapping from the corresponding semantic factor to word, therefore Nw x Ns.

Special case for A{1} because it does not need semantic information. Therefore A{1} is a Nw x 1 matrix. A{1}(one more) = A{1}(that) = 0.5

A{2}(index, agent) = 1, where index is calculated by finding the dictionary entry for the corresponding agent. E.g. the 3^rd^ agent state, ‘card J’, translates to ‘joker’ in the dictionary, which is at the 5^th^ entry. Therefore A{2}(5, 3) = 1. The rest of semantic-lemma mappings are defined in the same fashion.

When one semantic value corresponds to multiple lemma entries, e.g. the patient ‘tennis’ (1^st^ patient) can be translated into either ‘the tennis’ (11^th^ lemma entry), or ‘the game’ (13^th^), we give higher probability to the “meaning 1” mapping, e.g. A{4}(11, 1) = 0.8, A{4}(13, 1) = 0.2.
