# Supplementary material for: A deep hierarchy of predictions enables online meaning extraction in a computational model of human speech comprehension
Source: PLoS Biol. 2023 Mar 22;21(3):e3002046. doi: 10.1371/journal.pbio.3002046 (PMC10079236; doi:10.1371/journal.pbio.3002046)
Supplement: S2 Appendix — (DOCX) [file pbio.3002046.s007.docx]

# S2 Appendix: lemma-semantic mapping in the model’s mental lexicon

**Table 1. Mental lexicon of the model**

|  | lemma | Meaning 1 | Meaning 2 | Meaning 3 |
| --- | --- | --- | --- | --- |
| 1 | 'one more' | 'extra' | [] | [] |
| 2 | 'that' | 'that' | [] | [] |
| 3 | 'ace' | 'card A' | 'serve' | [] |
| 4 | 'sprint' | 'run' | [] | [] |
| 5 | 'joker' | 'card J' | [] | [] |
| 6 | 'tie' | 'neckband' | 'score' | [] |
| 7 | 'noise' | 'buzz' | [] | [] |
| 8 | 'wins' | 'win' | [] | [] |
| 9 | 'ruined' | 'ruin' | [] | [] |
| 10 | 'is' | 'be' | [] | [] |
| 11 | 'the tennis' | 'tennis' | [] | [] |
| 12 | 'the poker' | 'poker' | [] | [] |
| 13 | 'the game' | 'game' | 'tennis' | 'poker' |
| 14 | 'the evening' | 'evening' | [] | [] |
| 15 | 'enough' | 'sufficient' | [] | [] |
| 16 | 'surprising' | 'unexpected' | [] | [] |
| 17 | 'ugly' | 'not pretty' | [] | [] |
| 18 | 'unfair' | 'not fair' | [] | [] |
| 19 | 'loud' | 'high volume' | [] | [] |
| 20 | 'sharp' | 'high freq' | [] | [] |
