# Supplementary material for: A deep hierarchy of predictions enables online meaning extraction in a computational model of human speech comprehension
Source: PLoS Biol. 2023 Mar 22;21(3):e3002046. doi: 10.1371/journal.pbio.3002046 (PMC10079236; doi:10.1371/journal.pbio.3002046)
Supplement: S3 Appendix — (DOCX) [file pbio.3002046.s008.docx]

# Full expression of free energy and gradient descent algorithm for the top-level model (L_1_ and L_2_)

The expression of free energy (eq.16 in the main text) can be parameterized with respect to the posterior estimates of L_1_ and L_2_ factors

$$F=\sum_{\tau,k} syn_{k}^{\tau}\ln\left( syn_{k}^{\tau} \right)+\sum_{\alpha,j} s_{j}^{\alpha}\ln s_{j}^{\alpha}+\sum_{m} c_{m}\ln c_{m}+\sum_{a} ST_{a}\ln ST_{a}$$

$$-\sum_{i,j,k,\tau} W_{i}^{\tau}\ln A_{i,j,k}^{\left( k \right)}syn_{k}^{\tau}s_{j}^{\alpha\left( k \right)}-\sum_{a,k,\tau} \ln Z_{k,a}^{\tau}syn_{k}^{\tau}ST_{a}$$

$$-\sum_{j,a,m,\alpha} \ln L_{j,m,a}^{\left( \alpha\right)}s_{j}^{\alpha}c_{m}ST_{a}-\sum_{a} \ln H_{a}ST_{a}-\sum_{m} \ln D_{m}c_{m}$$

We can then derive partial derivatives of F with respect to Q:

$$\frac{\partial F}{\partial syn_{k}^{\tau}}=\ln syn_{k}^{\tau}-\sum_{i,j} W_{i}^{\tau}s_{j}^{\alpha\left( k \right)}\ln A_{i,j,k}^{\left( k \right)}-\sum_{a} ST_{a}\ln Z_{k,a}^{\left( \tau\right)}, \tau= 1,\cdots,n$$

$$\frac{\partial F}{\partial s_{j}^{\alpha}}=\ln s_{j}^{\alpha}-\sum_{i} \left( W_{i}^{2}syn_{k}^{2}+\cdots+W_{i}^{n}syn_{k}^{n} \right)\ln A_{i,j,k}^{k\left( \alpha\right)}-\sum_{m,a} c_{m}ST_{a}\ln L_{j,m,a}^{\alpha}, \alpha= \{A,R,P,M \}$$

$$\frac{\partial F}{\partial c_{m}}=\ln c_{m}-\sum_{j,a} s_{j}^{\left( 1 \right)}ST_{a}\ln L_{j,m,a}^{\left( 1 \right)}-\cdots-\sum_{j,a} s_{j}^{\left( n_{s} \right)}ST_{a}\ln L_{j,m,a}^{\left( n_{s} \right)}-\ln D_{m}$$

$$\frac{\partial F}{\partial ST_{a}}=\ln ST_{a}-\sum_{k} syn_{k}^{\left( 1 \right)}\ln Z_{k,a}^{\left( 1 \right)}-\cdots-\sum_{k} syn_{k}^{\left( n \right)}\ln Z_{k,a}^{\left( n \right)}-\sum_{j,m,\alpha} \ln L_{j,m,a}^{\left( \alpha\right)}s_{j}^{\alpha}c_{m}-\ln H_{a}$$

To solve the above equations, we follow Friston et al. (1) and define an auxiliary variable *v* for the estimation of each factor *x*. Let *x* ≡ σ(*v*), where σ() denotes the softmax function. We can then solve *v* and *x* using gradient descent:

$$\dot{v_{k}}=-\frac{\partial F}{\partial x_{k}}$$

With this choice for $\dot{v_{k}}$, the update equations are

$$v_{k}\left( t+\Delta t \right)\approx v_{k}\left( t \right)-\Delta t\frac{\partial F}{\partial x_{k}}$$

$$\vec{x}\left( t+\Delta t \right)\equiv\vec{\sigma}\left( \vec{v}\left( t+\Delta t \right) \right)\approx\vec{\sigma}\left( v_{k}\left( t \right)-\Delta t\frac{\partial F}{\partial x_{k}} \right)$$

# Reference

1. Friston KJ, Parr T, de Vries B. The graphical brain: Belief propagation and active inference. Netw Neurosci. 2017;1(4):381-414.
